# IGF1/IGF-1R promotes hepatocellular carcinoma progression by activating the Akt/GSK-3β pathway

**DOI:** 10.1371/journal.pone.0346926

**Published:** 2026-04-09

**Authors:** Jiaojiao Liang, Xueyi Song, Yang Liu, Guoyu Yang, Bairu Zhu, Xiaolong Tang

**Affiliations:** 1 Maternal and child health Hospital of Huai nan, The Sixth People’s Hospital of Huainan, Huainan, China; 2 Medical School, Anhui University of Science &Technology, Huainan, China; Universita degli Studi della Campania Luigi Vanvitelli, ITALY

## Abstract

The incidence of hepatocellular carcinoma (HCC) is increasing each year, with challenges such as increasing drug resistance and a high post-treatment recurrence rate. Therefore, investigating the novel pathogenic mechanisms is warranted. In this study, we investigated novel molecular mechanisms that affect HCC progression. Immunofluorescence analysis, immunohistochemical staining, and immunoblotting were performed to assess elevated IGF-1R expression in HCC cells. The EdU incorporation and colony formation assays revealed that IGF-1R promotes HCC cell proliferation. Furthermore, wound healing and Transwell migration assays revealed that IGF-1R phosphorylation enhances the migration of HCC cells. In addition, JC-1 apoptosis assays revealed that IGF-1R inhibits HCC cell apoptosis. Immunoblotting was performed to assess the protein phosphorylation level of Akt/GSK-3β downstream of IGF1/IGF-1R to explore the molecular mechanism. IGF-1R expression was significantly increased in HCC cells, and ligand-induced phosphorylation promoted HCC cell proliferation and migration and inhibited apoptosis. Additional studies revealed that the activation of IGF-1R phosphorylation promotes epithelial–mesenchymal transition in HCC cells by increasing the phosphorylation levels of Akt and GSK-3β. Collectively, our study findings suggest that IGF-1/IGF-1R promotes HCC progression by activating the Akt/GSK-3β pathway.

## Introduction

Hepatocellular carcinoma (HCC) is a type of malignant tumor whose incidence has been increasing over the past few decades; this has imposed a substantial societal and economic burden. Several risk factors contribute to the high incidence of HCC. While hepatitis B and C viruses are decreasing as predisposing factors, metabolic dysfunction-associated fatty liver disease is increasingly being recognized as a risk factor [1]. Recently, HCC has become increasingly resistant to treatment with many chemotherapeutic agents, including sorafenib [2]. Postoperative recurrence imposes substantial clinical and socioeconomic burdens. Although studies on liver cancer treatments and molecular mechanisms are constantly being updated, the therapeutic outcomes remain inferior for most patients. Therefore, identifying new molecular mechanisms for HCC progression and new therapeutic targets is warranted.

Recent national and international studies have revealed that the insulin-like growth factor 1 (IGF1)/insulin-like growth factor 1 receptor (IGF-1R) axis is closely associated with HCC pathogenesis. IGF1 is a polypeptide hormone that acts via its specific receptor, IGF-1R, which is located on the cell membrane. Under normal physiological conditions, the IGF1/IGF-1R signaling pathway plays a role in regulating cell growth, proliferation, and differentiation. However, the aberrant activation of this pathway is closely associated with HCC development. Studies have revealed that IGF1 and IGF-1R expression is significantly increased in HCC tissues. Overactivation of the IGF1/IGF-1R pathway results in dysregulated cell signaling, promotes tumor cell proliferation, and enhances resistance to apoptosis [3]. In particular, the activation of the Akt/GSK-3β pathway plays an essential role in HCC [4–5]. Protein kinase B (Akt) is a key regulator of cell survival signaling [6]. Akt activation via Thr308 and Ser473 phosphorylation represents a central downstream regulatory mechanism of the IGF1/IGF-1R signaling pathway. Glycogen synthase kinase-3 (GSK-3), a widely expressed protein kinase, comprises both GSK-3α and GSK-3β isoforms. Akt phosphorylates the Ser21 residue of GSK-3α and the Ser9 residue of GSK-3β, thereby inhibiting their enzymatic activities [7]. Epithelial–mesenchymal transition (EMT) is a vital factor in HCC progression. It is characterized by E-cadherin loss and N-cadherin acquisition. This transition enables HCC cells to become more mobile, allowing them to penetrate the basement membrane and enter the bloodstream or lymphatic system, thereby facilitating the development of distant metastases. EMT is associated with the increased proliferation of tumor cells [8–9] and is closely related to HCC resistance to chemotherapy [10]. EMT activates transcription factors such as Snail, Twist, and ZEB1/2, which downregulate epithelial properties and upregulate mesenchymal properties, thereby facilitating the invasion of cancer cells into surrounding tissues. Snail, a transcriptional repressor of E-cadherin, is frequently overexpressed in HCC and is associated with poor prognosis [11]. GSK-3β promotes progression and chemoresistance in several cancers, including colorectal cancer, by promoting cancer progression and chemotherapy resistance [12–14]. GSK-3β serves as a downstream target of the Akt signaling pathway, promoting EMT by facilitating drug resistance and migration capacity, thereby facilitating the transformation of epithelial cells into mesenchymal cells [15–17].

In the present study, we confirmed high IGF-1R expression in HCC cells and demonstrated that IGF-1R triggers the proliferation, migration, and anti-apoptotic effects of HCC cells. Meanwhile, IGF-1R promotes EMT in HCC by activating the Akt/GSK-3β pathway. These results suggest that IGF1/IGF-1R promotes HCC progression by activating the Akt/GSK-3β pathway. Our study findings provide a reference for exploring novel molecular mechanisms underlying HCC progression and identifying new therapeutic targets.

## Materials and methods

### Cell culture and reagents

The HCC cell lines HepG2 and Hep3B were obtained from Xiamen Yimo Biotechnology Co., Ltd (China). The normal human hepatocyte line HHL-5 was purchased from Shanghai Qingqi Biotechnology Co., Ltd. Cells were cultured in Gibco medium supplemented with 10% fetal calf serum (FBS, Zhejiang Tianhang Biotechnology Co., Ltd.) at 37°C and 5% CO_2_. The lentiviral vectors were prepared by Shanghai Biological Engineering Technology Co., Ltd. Antibodies against IGF-1R, p-IGF-1R, Akt, phospho-Akt (Ser473, p-Akt), β-actin, GSK-3β, p-GSK-3β (Ser9), E-cadherin, N-cadherin, and snail were purchased from Cell Signaling Technology (Danvers, MA, USA). The EdU reagent kit was purchased from Shanghai Beyotime Company, and the JC-1 reagent was purchased from Sigma-Aldrich (St. Louis, MO, USA).The study was approved by the ethics committees of Huainan Maternal and Child Health Hospital and Anhui University of Science and Technology (202401005).

### Lentiviral transfection

First, cells were seeded into 24-well plates and cultured until they reached 80% confluency. Then, the medium was replaced with 250 µl of lentivirus-containing medium (MOI = 25), followed by incubation for 4 h. After incubation for an additional 12 h, the cells were passaged in fresh culture flasks. Three siRNA constructs (siRNA1, siRNA2, and siRNA3) targeting distinct regions of the IGF-1R mRNA were generated. Each siRNA was designed to bind to a unique sequence in the IGF-1R transcript, ensuring specificity and minimizing the risk of off-target effects. Bioinformatic analysis was performed to select the target regions to maximize knockdown efficiency and specificity. However, comprehensive off-target analysis was not conducted. The siRNA sequences were designed using bioinformatics tools such as siDirect to minimize off-target effects. Western blotting revealed that siRNA2 and siRNA3 lentiviruses downregulated IGF-1R in HepG2 and Hep3B cells. Therefore, we randomly selected siRNA2 for subsequent experiments.

### 5-Ethynyl-2’-deoxyuridine (EdU) cell proliferation assay

Cells (1 × 10^5^/well) were seeded into 24-well plates and incubated in an incubator at 37℃ under 5% CO_2_ conditions for 24 h. The culture medium was discarded, and EdU-594 (500 µl) was added to the plates to a final concentration of 10 µM, followed by a 2-hour incubation. The culture medium was discarded, and the cells were first washed with the culture medium and then with phosphate-buffered saline (PBS, three times), followed by fixation with 4% paraformaldehyde for 15 min. Thereafter, the cells were treated with 0.3% Triton X-100 for 10 min. The click reaction solution was prepared according to the instructions, and the cells were treated with 500 µl of this solution for 30 min. The cells were then washed three times with PBS, followed by staining with Hoechst-33342 for 10 min. The cells were washed three times with PBS. The red fluorescence ratio was determined under an inverted microscope to assess the cell proliferation ability.

### Cell clone formation assay

Cells (1,000 cells/well) were seeded into 6-well plates, followed by the addition of 2 ml of culture medium and incubation for 2 weeks. Subsequently, the medium was discarded, and the cells were washed three times with PBS, followed by fixation in 4% paraformaldehyde for 15 min. The washing step was repeated, followed by staining with crystal violet for 10 min. Finally, the number of cell clones was counted and photographed.

### Scratch healing assay

A marker was used to draw three horizontal lines on the back of a 6-well plate as a reference for labeling. The cells were seeded into each well. After waiting for the cells to reach the bottom of the 6-well plate, a wound was created by making scratches using a 10-μl pipette tip. The supernatant was removed, and the cells were washed three times with PBS to ensure the wound area was clean. Then, 2 ml of serum-free medium was added to support cell growth. This facilitates cell migration and scratch healing. Scratch healing was observed under an inverted microscope at 0 and 48 h. Cell migration was recorded, and the migratory ability of the cells was assessed.

### Transwell assay

An 80-μm diameter Transwell chamber was placed in a 24-well plate and prewarmed to 37℃. Then, 3.0 × 10^5^ cells were suspended in 100 μl of serum-free medium and injected into the upper chamber of the Transwell. Thereafter, 600 μl of culture medium supplemented with 10% FBS was added to the lower chamber, which contains the chemical factors that attract migratory cells. The 24-well plate was placed in an incubator for further cell culture. Residual cells at the top of the Transwell upper chamber were gently removed with a cotton swab. To accurately assess the number of migrated cells, they were fixed with 4% paraformaldehyde for 30 min and stained with 0.5% crystal violet solution for 15 min. After washing three times with PBS, the migrated cells were observed under a microscope, photographed, and documented. The experiments were repeated at least three times. Finally, the average value of the number of migrated cells was calculated.

### JC-1 assay

Cells (1.5 × 10^5^ cells/well) were seeded in a 24-well plate and cultured overnight. Then, the JC-1 working solution (250 µl per well) was added, followed by incubation for 15 min and washing three times with PBS. Because JC-1 accumulates in the mitochondria with normal membrane potential and produces red fluorescence, JC-1 exhibits green fluorescence scattered as monomers in the cytoplasm owing to reduced mitochondrial membrane potential in apoptotic cells. By detecting the ratio of red to green fluorescence of JC-1, changes in mitochondrial membrane potential can be evaluated. A high red/green fluorescence ratio indicates normal mitochondrial membrane potential and healthy cells. In contrast, a low red/green fluorescence ratio indicates a decreased mitochondrial membrane potential and a state of apoptotic or dysfunctional cells. The green and red fluorescence intensity was analyzed using ImageJ software. Then, the red/green fluorescence ratio was calculated for each cell, and the average value for the overall analysis was determined.

### Western blotting

Cells were subjected to trypsin digestion in a culture bottle. Then, proteins were extracted with RIPA buffer, protease inhibitors, and lysates in a 1.5 ml Eppendorf tube. The samples were heated in a 100℃ water bath for 20 min, and protein concentration was measured. Protein samples (25 μl) were injected into a comb groove containing 8% sodium dodecyl sulfate–polyacrylamide gel for electrophoresis. The gel was concentrated at 80 V for 30 min, followed by electrophoresis at 120 V for 90 min to separate the proteins. The proteins were transferred to a PVDF membrane (100 V, 400 mA). The PVDF membrane was blocked with a solution containing 5% bovine serum albumin (BSA) for 1 h. Thereafter, the membrane was incubated overnight at 4 °C with the primary antibody. The next day, the secondary antibody working solution was added, followed by a 1-hour incubation. Then, the sample was blocked with BSA for 1 hour, and it underwent three washes with 1 × TBST solution (each for 10 minutes). Finally, a chemiluminescence mixture was added. Imaging was performed using a chemiluminescence imaging system.

### Laser confocal microscopy

For immunofluorescence analysis, the cells were washed with PBS and fixed with acetone for 20 min. After blocking with hydrogen peroxide, the primary antibody was added and incubated overnight at 4°C. Thereafter, the cells were incubated with fluorescently labeled secondary antibodies for 60 min at 25℃. The cell nuclei were then stained with DAPI for 10 min. The sample was sealed on a glass slide, and an anti-fluorescence quencher was used. Then, the cells were mounted and overlaid before examination under a laser confocal microscope. The microscope was opened, and laser light was applied to the slide, with simultaneous analysis. The control software was started, and the system was initialized. Appropriate wavelengths were selected based on the fluorescent dyes, and the slides were focused and imaged. Multilayer scanning images were subjected to 3D reconstruction, and the images were acquired.

### Mouse tumor formation assay

Twenty NOD-SCID female mice (4–5 weeks old) were randomly selected and divided into five groups, with four in each group. HepG2 cells were transplanted into the dorsal region of mice. Tumor growth was regularly monitored, and tumor volume and mass were measured to comprehensively assess the effect of IGF-1R on HCC growth. The tumor volume formula was as follows: V = (L × W^2^)/2, where V is the volume, L is the length, and W is the width. The experiment was conducted over 8 weeks. The tumor size was examined every 2 weeks, and the vital status of the mice was observed. Specific animal welfare signs such as body temperature or weight changes, tumor size or appearance, and abnormal behaviors were monitored. At the end of the 8 weeks, all the experimental mice were sacrificed. No mice died before reaching euthanasia standards. The mice were sacrificed via isoflurane overdose. The euthanasia process was 3 min, with a 100% mortality rate. All experiments were conducted in accordance with the document “Guiding Opinions on the Kind Treatment of Laboratory Animals. Mice were also sacrificed in cases of abnormal behavior, physical signs, appearance, or other factors that may affect experimental results to prevent severe pain caused by unavoidable death.

### Statistical analysis

All experiments were repeated at least three times. Data were expressed as mean ± standard deviation. GraphPad Prism8 software was used to perform statistical analysis. Each dataset was subjected to a t-test or one-way analysis of variance. Between-group comparisons were made using a two-tailed Student’s t-test. Multigroup Comparisons were made using one-way analysis of variance followed by Tukey’s post-hoc test. The error bars represent the standard error of the mean (SEM). P < 0.05 was considered statistically significant. Results were considered statistically significant at *P < 0.05, **P < 0.01, and ***P < 0.001.

## Results

### Increased IGF-1R expression in HCC cells

**Fig 1:**
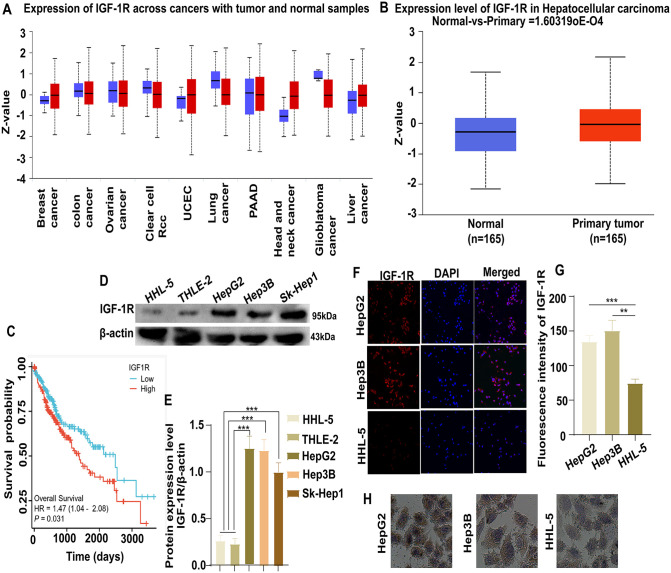
Increased IGF-1R expression in HCC cells. **(A)** IGF-1R expression in various cancers. **(B)** IGF-1R protein levels in patients with HCC and normal individuals (*P < 0.05). **(C)** Survival analysis of IGF-1R. **(D, E)** IGF-1R expression in HCC and normal liver cells (***P < 0.001). **(F, G)** Differences in protein expression between HCC and normal liver cells via laser confocal microscopy (**P < 0.01, ***P < 0.001). **(H)** Protein expression in HCC and normal liver cells via immunohistochemical staining. All data are expressed as mean ± standard deviation from at least three independent experiments. Comparisons between two groups were analyzed using a two-tailed Student’s t-test. Comparisons among multiple groups were analyzed via one-way analysis of variance followed by Tukey’s post-hoc test. A *P*-value of <0.05 was considered statistically significant.

The UALCAN database (https://ualcan.path.uab.edu/) was used to screen IGF-1R expression. It was high in breast cancer, uterine corpus endometrial carcinoma, head and neck cancer, and liver cancer (Figs 1A and 1B). Then, we performed Kaplan–Meier survival analysis to determine the prognostic value of IGF-1R in HCC. In The Cancer Genome Atlas dataset, we noted that patients with HCC and high IGF-1R mRNA expression had poor overall survival (*P =* 0.031, Fig 1C). Meanwhile, western blotting revealed significantly higher IGF-1R expression in HepG2 (*P* = 0.0005), Hep3B (*P* = 0.0002), and SK-Hep 1 (*P* = 0.0004) cells compared with THLE-2 and HHL-5 cells (Figs 1D and 1E). Therefore, we selected HepG2, Hep3B, and HHL-5 cells as the study subjects. Laser confocal microscopy revealed increased IGF-1R expression in HepG2 (*P =* 0.0007) and Hep3B (*P* = 0.0012) compared with HHL-5 cells (Figs 1F and 1G). Subsequently, immunohistochemical staining was performed to compare IGF-1R expression in HepG2, Hep3B, and HHL-5 cells. IGF-1R expression was strongly positive in the membranes of HCC cells (Fig 1H). Collectively, these results suggest the high expression of IGF-1R, indicating its role in HCC development.

### IGF-1R promotes HCC cell proliferation

**Fig 2:**
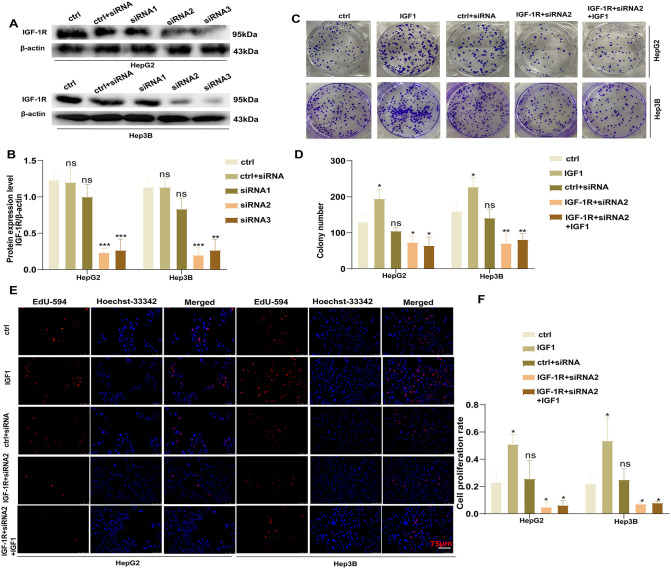
IGF-1R promotes HCC cell proliferation. **(A, B)** Western blotting assay revealing that siRNA2 and siRNA3 lentiviruses downregulated IGF-1R in HepG2 and Hep3B cells (**P < 0.01, ***P < 0.001). **(C, D)** Clone formation assay to detect the differences in the cell proliferation abilities of each cell group (*P < 0.05, **P < 0.01). **(E, F)** EdU assay to detect the differences in the cell proliferation abilities of each group (*P < 0.05, × 200). All data are expressed as mean ± standard deviation from at least three independent experiments. Comparisons between two groups were analyzed using a two-tailed Student’s t-test. Comparisons among multiple groups were analyzed via one-way analysis of variance followed by Tukey’s post-hoc test. A *P*-value of <0.05 was considered statistically significant. Data represent mean ± standard error of mean. n = 3.

To investigate the effect of IGF-1R on HCC progression, we used lentivirus-mediated siRNA targeting IGF-1R to knock down IGF-1R expression in HCC cells. Western blotting revealed that both siRNA2 and siRNA3 effectively downregulated IGF-1R expression in HepG2 cells (siRNA2 vs. Ctrl: *P* = 0.0002, siRNA3 vs. Ctrl: *P* = 0.0009) and Hep3B cells (siRNA2 vs. Ctrl: *P* = 0.0005, siRNA3 vs. Ctrl: *P* = 0.0014) (Figs 2A and 2B). Accordingly, siRNA2 was randomly selected for subsequent experiments. The experimental groups were as follows: the Ctrl + siRNA group served as the transfection control, and the Ctrl group was the untransfected group. Five experimental groups were established: Ctrl, IGF1 (100 ng/mL), Ctrl + siRNA, IGF-1R + siRNA2, and IGF-1R + siRNA2 + IGF1. The colony formation assay revealed significantly enhanced cell proliferation in the IGF1 (100 ng/mL) group compared with the Ctrl group (HepG2: *P* = 0.0107, Hep3B: *P* = 0.0314). In contrast, the proliferation rate was decreased in the IGF-1R + siRNA2 group compared with the Ctrl group (HepG2: *P* = 0.0135, Hep3B: *P* = 0.0027), Furthermore, IGF1 addition did not reverse this proliferation suppression in the IGF-1R + siRNA2 + IGF1 group (HepG2: *P* = 0.0344, Hep3B: *P* = 0.0014) (Figs 2C and 2D). Consistent findings were noted in the EdU assay, which revealed significantly increased cell proliferation in the IGF1 (100 ng/mL) group compared with the Ctrl group (HepG2: *P* = 0.0142, Hep3B: *P* = 0.0453). Meanwhile, the proliferation rate was reduced in the IGF-1R + siRNA2 group compared with the Ctrl group (HepG2: *P* = 0.0327, Hep3B: *P* = 0.0356) (Figs. 2E and 2F (×200)). Collectively, these results suggest that IGF-1R promotes HCC cell proliferation, which can be activated by stimulating IGF1.

### IGF-1R drives HCC cell migration and inhibits apoptosis

**Fig 3:**
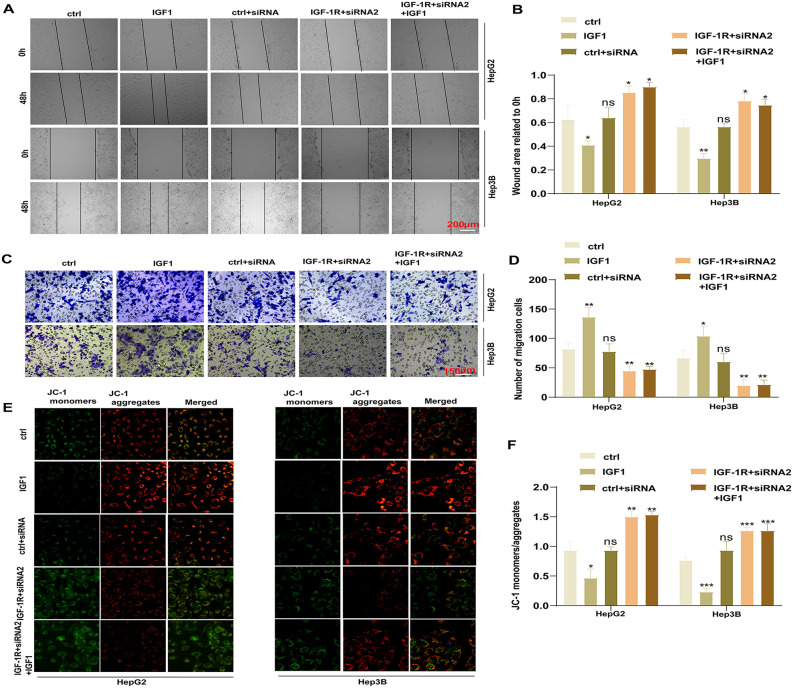
IGF-1R drives HCC cell migration and inhibits apoptosis. **(A, B)** Scratch healing assay to detect the differences in the cell migratory ability of each group (*P < 0.05, **P < 0.01). **(C, D)** Transwell assay to detect the differences in the cell migratory ability of each group (*P < 0.05, **P < 0.01). **(E, F)** JC-1 assay to detect the differences in the apoptotic ability of each group (*P < 0.05, **P < 0.01, ***P < 0.001). All data are expressed as mean ± standard deviation from at least three independent experiments. Comparisons between two groups were analyzed using a two-tailed Student’s t-test. Comparisons among multiple groups were analyzed using one-way ANOVA followed by Tukey’s post-hoc test. A *P*-value of <0.05 was considered statistically significant. Data represent mean ± SEM. n = 3.

To further investigate the role of IGF-1R in HCC progression, we performed wound healing assays. Would healing assays revealed a significant enhancement of cell migration in the IGF1 (100 ng/mL) group compared with the Ctrl group (HepG2: *P* = 0.0373, Hep3B: *P* = 0.0032). In HepG2 and Hep3B cells transfected with IGF-1R siRNA2 lentivirus, the migratory ability was decreased in the IGF-1R + siRNA2 group compared with the Ctrl group (HepG2: *P* = 0.0384, Hep3B: *P* = 0.0111). The subsequent addition of IGF1 did not restore the cell migration ability in the IGF-1R + siRNA2 group (Figs 3A and 3B). Similar findings were observed in the Transwell assays, with a significant increase in cell migration in the IGF1 (100 ng/mL) group compared with the Ctrl group (HepG2: *P* = 0.0080, Hep3B: *P* = 0.0394). Furthermore, the migratory ability was decreased in the IGF-1R + siRNA2 group compared with the Ctrl group (HepG2: *P* = 0.0088, Hep3B: *P* = 0.0078) (Figs 3C and 3D).

Next, to determine the effect of IGF-1R on HCC cell apoptosis, we performed JC-1 assays. They revealed that the cell apoptotic rate was decreased in the IGF1 group compared with the Ctrl group (HepG2: *P* = 0.0201, Hep3B: *P* = 0.0003). However, in cells transfected with IGF-1R siRNA2, the apoptotic rate was enhanced in the IGF-1R + siRNA2 group compared with the Ctrl group (HepG2: *P* = 0.0058, Hep3B: *P* = 0.0004). IGF1 treatment did not reverse this pro-apoptotic effect in the IGF-1R + siRNA2 + IGF1 group (Figs 3E and 3F). Collectively, these results suggest that IGF-1R inhibits the apoptosis of HCC cells, which is activated by IGF1 stimulation.

### IGF-1R drives HCC progression via the Akt/GSK-3β signaling pathway

**Fig 4:**
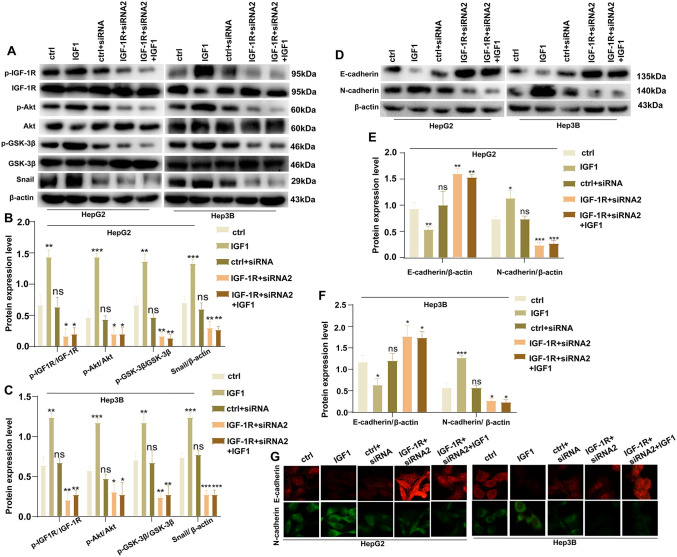
IGF-1R drives HCC progression via the Akt/GSK-3β signaling pathway. (A, B, C) Immunoblotting assay to detect the levels of p-IGF-1R, p-Akt, p-GSK-3β, and Snail in each cell group (*P < 0.05, **P < 0.01, ***P < 0.001). (D, E, **F)** Immunoblotting assay to detect the protein levels of E-cadherin and N-cadherin in each cell group (*P < 0.05, **P < 0.01, ***P < 0.001). Each immunoblotting experiment included three independent biological replicates. Band intensity was quantified using ImageJ software. Data were expressed as mean ± SEM. β-actin was used as the loading control. **(G)** Immunofluorescence staining assay to detect the protein levels of E-cadherin and N-cadherin in each cell group. Comparisons between two groups were analyzed using an unpaired t-test. Data were derived from three independent experiments (n = 3 biological replicates/group). Error bars represent SEM, and statistical significance is indicated in the figures.

To understand the molecular mechanisms by which IGF-1R affects HCC progression, we performed immunoblotting experiments in HepG2 and Hep3B cells. The protein levels of p-IGF-1R (HepG2: *P* = 0.0023, Hep3B: *P* = 0.0013), p-Akt (HepG2: *P* = 0.0002, Hep3B: *P* = 0.0002), p-GSK-3β (HepG2: *P* = 0.0018, Hep3B: *P* = 0.0061), and Snail (HepG2: *P* = 0.0007, Hep3B: *P* = 0.0004) were significantly increased in the IGF1 group compared with the Ctrl group. In contrast, transfection with IGF-1R siRNA2 lentivirus decreased the levels of p-IGF-1R (HepG2: *P* = 0.0106, Hep3B: P = 0.0080), p-Akt (HepG2: *P* = 0.0390, Hep3B: P = 0.0161), p-GSK-3β (HepG2:*P* = 0.026, Hep3B: *P* = 0.0022), and Snail (HepG2: *P* = 0.0080, Hep3B: *P* = 0.0006) in the IGF-1R + siRNA2 group compared with the Ctrl group. The subsequent addition of IGF1 did not restore the protein levels of p-IGF-1R, p-Akt, p-GSK-3β, and Snail in the IGF-1R + siRNA2 + IGF1 group (Figs 4A-4C). Meanwhile, E-cadherin levels decreased in the IGF1 group compared with the Ctrl group (HepG2: *P* = 0.0058, Hep3B: *P* = 0.0129), increased in the IGF-1R + siRNA2 group compared with the Ctrl group (HepG2: *P* = 0.0016, Hep3B: *P* = 0.0242), and remained elevated in the IGF-1R + siRNA2 + IGF1 group (HepG2: *P* = 0.0013, Hep3B: *P* = 0.0105). However, N-cadherin and E-cadherin levels exhibited an opposite trend in each group. N-cadherin levels were increased in the IGF1 group compared with the Ctrl group (HepG2: *P* = 0.0132, Hep3B: *P* = 0.0007); in contrast, they decreased in the IGF-1R + siRNA2 group compared with the Ctrl group (HepG2: *P* = 0.0004, Hep3B: *P* = 0.0158). Similarly, N-cadherin levels decreased in the IGF-1R + siRNA2 + IGF1 group (HepG2: *P* = 0.0003, Hep3B: *P* = 0.0111) (Figs 4D-4F).

Immunofluorescence staining further confirmed that E-cadherin expression was lower in the IGF1 group than in the Ctrl group and higher in the IGF-1R + siRNA2 group than in the Ctrl group. Furthermore, E-cadherin expression was increased in the IGF-1R + siRNA2 + IGF1 group, whereas N-cadherin expression exhibited the opposite pattern (Fig 4G). Collectively, these results suggest that IGF-1R promotes the proliferation, migration, and resistance to apoptosis of HCC cells by activating the Akt/GSK-3β signaling pathway, and that it is involved in EMT progression in HCC.

### Reversal of IGF-1 affects HCC by suppressing the Akt/GSK-3β pathway

**Fig 5:**
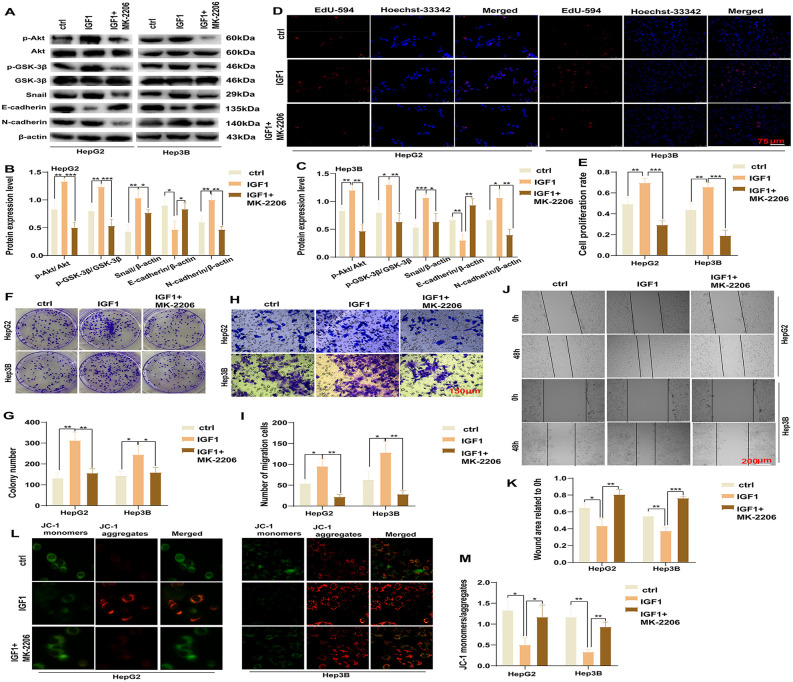
Reversal of IGF-1R’s Effects on HCC by suppressing Akt/GSK-3β (A, B,C). The protein levels of p-Akt, p-GSK-3β, Snail, E-cadherin, and N-cadherin across experimental groups (*P < 0.05, **P < 0.01, ***P < 0.001). **(D, E)** Differences in the proliferation capacity of each group via the EdU assay (**P < 0.01, ***P < 0.001). **(F, G)** Cell proliferation ability of each cell group in the clone formation assay (*P < 0.05, **P < 0.01). (H, **I)** Transwell assay to determine the cell migration ability of each group (*P < 0.05, **P < 0.01). **(J, K)** Cell migration ability of each group. **(L, M)** Apoptotic capacity of each group via the JC-1 assay (*P < 0.05, **P < 0.01). All data are expressed as mean ± standard deviation from at least three independent experiments. Comparisons between two groups were analyzed using a two-tailed Student’s t-test. Comparisons among multiple groups were analyzed via one-way analysis of variance followed by Tukey’s post-hoc test. A P-value of <0.05 was considered statistically significant. Error bars represent SEM, and statistical significance is indicated in the figures.

To validate the role of the Akt/GSK-3β signaling pathway in HCC regulation, we treated HepG2 and Hep3B cells with the Akt phosphorylation inhibitor MK-2206. After adding the inhibitor (IGF1 + MK-2206 group), compared with the IGF1 group, the initially increased protein levels of p-Akt (HepG2: *P* = 0.0002, Hep3B: *P* = 0.0011), p-GSK-3β (HepG2: *P* = 0.0007, Hep3B: *P* = 0.0032), Snail (HepG2: *P* = 0.0474, Hep3B: *P* = 0.0101), and N-cadherin (HepG2: *P* = 0.0013, Hep3B: *P* = 0.0032) were decreased, whereas the originally decreased levels of E-cadherin (HepG2: *P* = 0.0295, Hep3B: *P* = 0.0020) were increased (Figs 5A-5C). These results support the hypothesis that IGF-1R promotes HCC progression by activating the Akt/GSK-3β signaling pathway.

Subsequently, we performed the EdU assays to evaluate the effects of IGF-1R on the proliferative capacity of HCC cells. The Akt phosphorylation inhibitor MK-2206 significantly reversed the promotive effect of IGF1 on the proliferation of both HepG2 (IGF1 + MK-2206 vs. IGF1: *P* = 0.0003) and Hep3B cells (IGF1 + MK-2206 vs. IGF1: *P* = 0.0002). Colony formation assays further confirmed this finding (IGF1 + MK-2206 vs. IGF1; HepG2: *P* = 0.0040, Hep3B: *P* = 0.0374) (Figs 5D-5G). Furthermore, Transwell assays revealed that the Akt phosphorylation inhibitor MK-2206 significantly reversed the migration-promoting effects of IGF1 in HepG2 (IGF1 + MK-2206 vs. IGF1: *P* = 0.0025) and Hep3B cells (IGF1 + MK-2206 vs. IGF1: *P* = 0.0036). Consistent findings were noted in the scratch healing assays (IGF1 + MK-2206 vs. IGF1; HepG2: *P* = 0.0036, Hep3B: *P* = 0.0001) (Figs 5H-5K). Meanwhile, MK-2206 also reversed the anti-apoptotic effects of IGF1 in HepG2 (IGF1 + MK-2206 vs. IGF1: *P* = 0.0265) and Hep3B cells (IGF1 + MK-2206 vs. IGF1: *P* = 0.0013) (Figs 5L and 5M). Collectively, these results suggest that IGF-1R mediates the biological effects on HCC cell proliferation, migration, and apoptosis via the Akt/GSK-3β signaling pathway.

### Effect of IGF-1R on HCC progression in vivo

**Fig 6:**
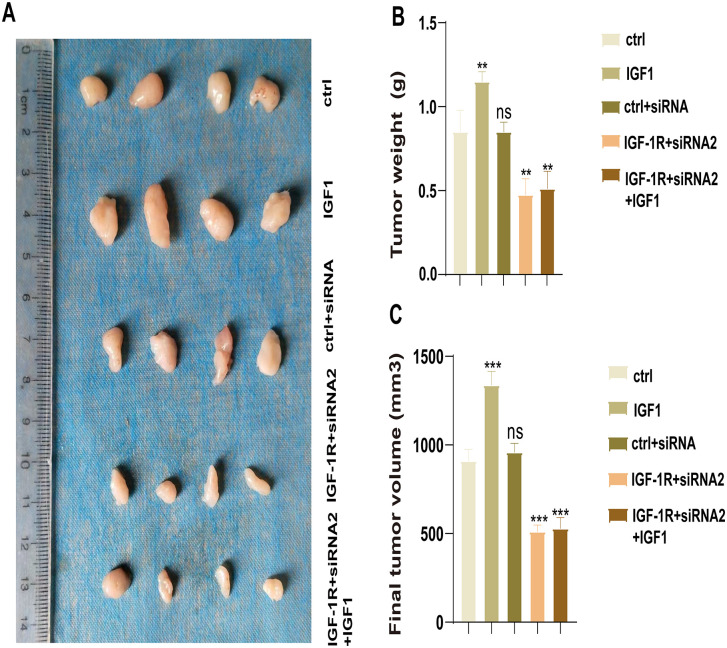
Effect of IGF-1R on HCC growth in vivo. (A, B, **C)** Tumor weight and volume formed by each group of cells in vivo (**P < 0.01, ***P < 0.001). All data are expressed as mean ± standard deviation (SD) from at least three independent experiments. Comparisons between two groups were analyzed using a two-tailed Student’s t-test. In contrast, comparisons among multiple groups were analyzed using one-way analysis of variance, followed by Tukey’s post hoc test. A P-value of <0.05 was considered statistically significant.

To verify the effect of IGF-1R on HCC growth, we performed in vivo tumor formation experiments. First, HepG2 cells were subcutaneously injected into mice; then, the tumor growth parameters (weight and volume) were monitored and quantified. We observed that the tumor weight formed by the cells in the IGF1 group was significantly higher than that formed by cells in the Ctrl group (*P* = 0.0054). The tumor weight was decreased in the IGF-1R + siRNA2 group compared with the Ctrl group (*P* = 0.0034), whereas the tumor weight was comparable between the IGF-1R + siRNA2 + IGF1 and IGF-1R + siRNA2 groups (*P* = 0.6131) (Figs 6A and 6B). The final tumor volume exhibited the same trend. The final tumor volume was significantly larger in the IGF1 group than in the Ctrl group (*P* = 0.0002). The tumor volume was decreased in the IGF-1R + siRNA2 group compared with the Ctrl group. However, the IGF-1R + siRNA2 + IGF1 and IGF-1R + siRNA2 groups did not exhibit significant differences (*P* = 0.6353) (Fig 6C). These findings suggest that IGF-1R promotes HCC cell growth in mice and that IGF1 activates it. This finding is consistent with our hypothesis, further validating the vital role of IGF1/IGF-1R in regulating HCC progression.

## Discussion

HCC is the sixth most common tumor type globally [18]. The high mortality rate and increased resistance to commonly used anticancer drugs such as sorafenib are considered major therapeutic challenges for patients with advanced HCC [19–20]. Therefore, elucidating the detailed mechanisms underlying HCC occurrence and development is vital. In the present study, we confirmed that IGF-1R expression is increased in HCC cells, as well as its role in promoting the proliferation of HCC cells and inhibiting apoptosis via the Akt/GSK-3β signaling pathway.

IGF-1R acts as a membrane surface receptor. Upon binding to its ligands IGF-1 or IGF-2, it activates downstream signaling pathways involved in biological effects, including the MAPK, PI3K/Akt, and JAK/STAT pathways, by regulating them to participate in the proliferation, migration, anti-apoptosis, and EMT of tumor cells [21–23]. IGF-1R is highly expressed in various tumors, including triple-negative breast cancer and HCC [24–25]. It can function as an intermediate signaling molecule. miR-486-5p can inhibit HCC progression by inhibiting IGF-1R and its downstream mediators mTOR, STAT3, and C-MYC [26]. Furthermore, IGF-1R can promote HCC growth and invasion by activating the STAT3/Midkine/STAT3 signaling loop [27]. In addition, IGF-1R contributes to chemoresistance in HCC. The simultaneous blocking of the MAPK and PI3K/Akt signaling pathway with IGF-1R inhibitors, and combined with regorafenib, can be a more potent approach for treating HCC [28]. IGF-1R can regulate HCC sensitivity to sorafenib via the PI3K/Akt and RAS/raf/ERK signaling pathways [29]. In the present study, using immunofluorescence staining, immunohistochemical staining, and protein immunoblotting, we confirmed that IGF-1R expression was significantly higher in HCC lines than in normal hepatocyte cells. To elucidate the molecular mechanism by which IGF-1R promotes the proliferation of HCC cells, we designed and prepared three siRNA lentivirus with different targets for IGF-1R to inhibit IGF-1R gene expression and confirmed the results in HCC cells. We observed that siRNA2 and siRNA3 vector lentivirus exerted a strong inhibitory effect on IGF-1R expression in HepG2 and Hep3B cells. Subsequently, we randomly selected siRNA2 as the interfering lentivirus for IGF-1R to further investigate the molecular mechanism underlying IGF-1R in HCC development.

Akt is an important signaling molecule, and its downstream targets include mTOR, YAP, and GSK-3β [30–32]. The Phosphorylation activation of Akt is closely associated with the development of various cancers [33]. In the present study, using protein immunoblotting experiments, we noted that after applying siRNA2 to downregulate IGF-1R expression in HCC cells, the protein levels of p-Akt, p-GSK-3β, and Snail were also decreased, further inhibiting the proliferation and anti-apoptotic effects of HCC cells. Furthermore, EMT was suppressed in HCC cells after interference with IGF-1R. In addition, the application of the Akt inhibitor MK-2206 [34] effectively reversed the levels of p-Akt, p-GSK-3β, and Snail, simultaneously reversed the proliferation inhibition and pro-apoptotic effects of HCC cells with low IGF-1R levels, and reversed the EMT transformation of HCC cells. Moreover, using animal experiments, we verified that IGF-1R promotes HCC progression, which is affected by IGF1 activation. Collectively, these results suggest that the Akt/GSK-3β signaling cascade plays a vital role in the proliferation and anti-apoptotic process of HCC cells and is regulated by activating IGF-1R.

In conclusion, although previous studies have revealed that IGF-1R silencing via siRNA significantly decreased p-Akt/p-GSK-3β levels, treatment with MK-2206 can reverse the EMT phenotype caused by the phosphorylation activation of IGF-1R. These findings confirm that Akt/GSK-3β are vital signaling nodes downstream of IGF-1R. However, the precise regulatory mechanism governing GSK-3β remains unclear. Our study data suggest a novel mechanism by which IGF-1/IGF-1R drives EMT via Akt/GSK-3β signaling. Unlike previous studies that have focused on PI3K/Akt [35], we elucidated the novel role of GSK-3β in IGF-1R-mediated EMT. For example, we demonstrated that IGF-1/IGF-1R drives HCC progression via the Akt/GSK-3β pathway and that IGF-1R induces the EMT process via the Akt/GSK-3β pathway in HCC, suggesting a potential therapeutic target for anti-HCC therapy.

In the future, we will investigate whether the role of IGF-1R in HCC EMT contributes to therapy resistance, with a focus on elucidating its crosstalk with immune checkpoints, such as PD-L1, and its impact on angiogenesis and immune escape, to identify novel therapeutic targets. Considering the established role of PD-L1 in HCC immune evasion, we will specifically investigate if IGF-1R silencing regulates PD-L1 expression via the Akt/GSK-3β pathway.

## Supporting information

S1 File Original result images including Western blot images. (7Z)

S2 File Statistical charts of duplicate data for all images to facilitate reproducibility in experimental studies. (7Z)

S3 File Statistical data for all images, including statistical methods. (7Z)
